# Laparotomy for Intestinal Obstruction, Mode of Operating

**Published:** 1885-09

**Authors:** J. Greig Smith

**Affiliations:** Surgeon to the Bristol Royal Infirmary


### ABSTRACT FROM A CLINICAL LECTURE ON FIVE CASES OF LAPAROTOMY FOR INTESTINAL OBSTRUCTION: THE MODE OF OPERATING.*

BY J. GREIG SMITH, M. A., F. R. S. E., SURGEON TO THE BRISTOL ROYAL INFIRMARY.

An abdominal section for obstruction of the bowels is usually a very different proceeding from one for removal of a tumor. In the former case, the abdominal walls will be hard, tense and unyielding, and the intestines will be full of fluid and gas under considerable pressure. When the abdomen is opened, and pressure is partly removed, the gas expands, dilating the bowels, and forcing them through the wound. In ordinary cases of laparotomy, where the gut is flaccid and empty, it is easy enough to isolate and run our fingers along it for a considerable distance; in cases of obstruction, this is a matter of great difficulty. The gut, when distended, is too large to be easily grasped between the fingers; its walls are too thin and impalpable to be isolated by mere touch; and, if any degree of peritonitis be present, it will inconveniently stick to the fingers. The exploring hand at every step is caught in the loops of distended bowel, which wind about in most confusing manner inside the abdominal cavity. In fact, it is just when the physical conditions are most strongly against us that we have to look for the cause of an intestinal obstruction inside the abdomen.

A course of procedure has been laid down for our guidance in such cases, and it will be well for you always to follow this course.

*British Medical Journal.

Though I have not yet derived any help from it, it is not unlikely to be of good service in leading us to the seat of obstruction. The plan is as follows: First carry the hand to the cæcum; if it be found distended, the obstructing cause will probably be somewhere in the large bowel below this, and the hand is carried up the ascending colon, across the traverse colon, and down the descending colon as far as the sigmoid flexure, seeking for the stricture all the way. If the cause be not here, we are then told to carry the hand from left to right under the distended coils, seek for collapsed bowel, and follow it up till we meet with distension, when we shall probably find the cause. Now, it is much easier to lay down these directions than to put them into practice. If the small intestine be much distended with gas, we may have the traverse colon pushed up under the ribs; and to reach it there over coils of dilated gut, under hard, brawny abdominal walls, and, more than this, to isolate it and diagnose its condition, is an undertaking, to say the least of it, of very considerable difficulty. If we can find undilated bowel, we may follow it up readily enough; but how often do we expect to find empty bowel in these cases? I have not yet met with it. We must be prepared to find the bowel dilated everywhere, at some parts more than others, but nowhere collapsed; and we must be prepared to look for the obstruction amid the endless contortions of these soft, sticky and dilated coils.

As I have said, I have always observed these directions, but only to find the observance so much valuable time wasted. To proceed to examine, inch by inch, the whole length of bowel will undoubtedly be successful as far as the discovery of the site of obstruction is concerned; but not probably so as regards saving the life of the patient. In such a procedure, the amount of rough handling, and the length of time spent over it, must tell seriously against the chances of recovery.

The plan I would recommend to you is this: The most distended portion of bowel are usually nearest to the surface; move these about gently, and fix upon any part that appears to be more congested than another. Follow this part in the direction of increasing congestion down into the cavity wherever it may lead.

If now the cause be discovered, it may be at once treated. If not, I would then recommend a plan which has been almost universally condemned: that is to permit the bowel to extrude. The wound is covered with some layers of fine cloth, or better still, by a large flat sponge wrung out of warm carbolic or boracic lotion, and covered with gutta-percha tissue to prevent evaporation and cooling, and the most distended coils are coaxed out under this covering. Carefully watch the gut as it comes out. One end of the coil will be more distended than the other, and will come out less readily, and this end will probably be more injected. As they continue to extrude, these differences will be more marked, and we will be able satisfactorily to decide that one end of the coil is nearest the cause of obstruction. We follow this end wherever it leads; it will certainly lead us to our goal. I have three times followed this course, and have been charmed with its simplicity and efficiency.

Turning to the extruded coils of bowel, the bulk that they assume may look somewhat alarming. If the distention be not very great, you may try tc return them. Spread the hands over the warm antiseptic covering, gather the bowels together, and by steady, gentle, equable pressure send their contents into the bowels that lie inside the abdomen. When they are nearly emptied, and occupy about one-fourth of their previous bulk, they may be slipped through the wound, while an assistant, with finger hooked in at each extremity, pulls the opening forwards. The abdominal wall is, so to speak, pulled up over the bowels. A flat sponge is now laid over them to protect them and keep them in position while the wound is stitched up.

But it is by no means certain that we are always right in thus closing up an abdominal wound over distended bowels. In fact, I believe that in doing so we are nearly always wrong. I believe that this distention with fluid and gas is in itself a serious factor in the malady. It certainly acts as an obstruction. I place before you these coils of intestine, with the mesentery attached, just removed from a dead body. They are filled to distention with fluid, and confined within the walls of this dish. You notice that the gut forms acute flexures, and that, at each of these, the mesenteric side of the bowel is pushed in, and acts as a sort of valve, blocking the calibre of the gut. As with any other tube, if you bend the bowel acutely enough, you will block its passage. Inside the abdominal cavity, where the confinement is greater, the flexures are more numerous and more acute than those on the table, and you may easily satisfy yourself by experiment that the passage of its contents is even more difficult. Practically this fact explains why we are so often disappointed with the effect of tapping the bowel to permit the escape of gas or fluid. We empty it down to the first or second flexure, and no further.

Such distention also has an injurious effect on the bowels themselves. An intestine that has been overdilated for hours, or perhaps days, and that is probably paralyzed by opium, we should expect to contract on its contents no more than a dilated bladder under the same circumstances. If it cannot pass its contents along, the obstruction is to all intents and purposes unrelieved. We know that such increase of pressure inside the abdomen is partly from its physical effects on the diaphragm and partly, no doubt, through injury to the sympathetic ganglia, a cause of serious illness. When added to the effects of intestinal obstruction, its gravity is increased tenfold.

Now, it is a matter of constant experience that free vomiting greatly relieves the patient. I am one of those who do not lightly regard the experiences of our forefathers; and I cannot believe that the very general esteem in which, from the days of Hipocrates and Praxagoras, they held the use of emetics in obstruction was utterly misplaced. And quite recently we have had recommended strongly by Kussmaul the removal of intestinal gases and fluids in these cases by the use of the stomach-pump. Relief is always claimed for this procedure, and, in not a few cases, positive cure.

I certainly consider the removal of this fluid and gas from the bowels, after relief of the constriction, as the most important detail in treatment. If it is to be done, it must be done rapidly, for no time must be wasted in these operations. Tapping will not do, for the flow through such a trocar as we should dare to push through the intestinal wall would be far too slow. We must incise the bowel. The blade of an ordinary scalpel, making a wound about one-third of an inch in length, is pushed through the bowel transversely at the point furthest removed from the mesentery; and, while you hold the opening, removed as far as possible from the wound, over a receiver, an assistant presses the sides of the abdomen and squeezes out the intestinal contents. At first, probably there will be a rush of gas, and then fluid will follow, watery or fæcal, as the case may be. When the abdominal walls are flaccid and the intestines are nearly emptied, we may stitch up the wound in the bowel, return it, and close the abdomen.

You have just seen this proceeding carried out in one case. The patient was in about as bad a condition as one ever sees upon an operating table. His pulse could scarcely be felt, and certainly could not be counted. His intestines were everywhere of a bright, rosy red, covered in many places with patches of yellow lymph, and everywhere fully distended. Semi-purulent fluid flowed from the abdomen when the incision was made, and a good deal more was mopped out. The constriction, a mesenteric band, was discovered in the way described, and easily divided. The bowel was incised, and through the opening, while the abdomen was being kneaded, large quantities of gas and fluid escaped. A continuous suture of fine catgut accurately closed this opening; the bowel was easily returned; the abdominal cavity was mopped out, and the wound sutured in flaccid abdominal walls. You have seen the case recover with no more trouble than any other abdominal section; with less trouble certainly than most cases of herniotomy. One example may be more impressive than much advice. I am sure you will not soon forget the lessons which this case has taught us.

In conclusion, I venture to submit to you these rules for your guidance in opening the abdomen for the relief of acute intestinal obstruction.

1. Make the incision in the middle line below the umbilicus.

2. Fix upon the most dilated or the most congested part of the bowel that lies near the surface, and follow it with the fingers as a guide to the seat of obstruction.

3. If this fail, insert the hand, and carry it successively to the cæcum, the umbilicus, and the promontory of the sacrum.

4. If this again fail, draw the intestine out of the wound, carefully covering it, until increase of distention on congestion, or both in one of the coils, gives an indication that the stricture lies near.

5. If there be considerable distention of the intestines, evacuate their contents by incision, and suture the wound. Never consider an operation for intestinal obstruction inside the abdomen finished until the bowels are relieved from over-distention.

6. Be expeditious, for such cases suffer seriously from shock. The whole operation ought to be concluded in half an hour.

